# Aquaculture Pathogens and Antimicrobial Resistance: A One Health Perspective

**DOI:** 10.3390/ijms27188125

**Published:** 2026-09-12

**Authors:** Avani Anu Geetha, Mydhily Soorej, Elishia Alex, Dhruva Rajesh, Nikhila S. Babu, Fathima Ishani, Sreelekshmi Renjith, Athul Sajeev, Jithu Paul Jacob, Bipin G. Nair, Geetha Kumar, Aravind Madhavan, Pradeesh Babu

**Affiliations:** 1 Amrita School of Biotechnology, Amrita Vishwa Vidyapeetham, Kollam 690525, Kerala, India; avaniag@am.amrita.edu (A.A.G.); am.ls.u3bit23033@am.students.amrita.edu (M.S.); am.ls.u3bit23022@am.students.amrita.edu (E.A.); am.ls.u3bit23113@am.students.amrita.edu (D.R.); am.ls.u3bit23137@am.students.amrita.edu (N.S.B.); am.ls.u3bit23023@am.students.amrita.edu (F.I.); am.ls.u3bit23145@am.students.amrita.edu (S.R.); am.ls.r4bio24433@am.students.amrita.edu (A.S.); bipin@am.amrita.edu (B.G.N.); gkumar@am.amrita.edu (G.K.); aravindmi@am.amrita.edu (A.M.); 2 St Alberts College (Autonomous), Ernakulam 682018, Kerala, India; jithupaul@alberts.edu.in

**Keywords:** antimicrobial resistance, aquaculture, one health, horizontal gene transfer, antibiotic alternatives

## Abstract

Antimicrobial resistance (AMR) has emerged as a critical global health concern that transcends human, animal, and environmental boundaries. The extensive and often indiscriminate use of antibiotics for disease control and growth promotion has accelerated the evolution and spread of resistant pathogens within the aquaculture sector. This review examines aquaculture-associated AMR through a One Health framework. We explore the molecular mechanisms of resistance such as efflux pumps, enzymatic degradation, mobile genetic elements, and horizontal gene transfer that drive the dissemination of antimicrobial resistance genes among aquatic microorganisms. The review synthesizes evidence linking AMR in aquaculture to public health impacts, including the transmission of resistant bacteria through seafood and environmental exposure. Current regulatory measures and stewardship policies are assessed to identify gaps in implementation, particularly in developing regions where enforcement and public awareness remain limited. Review highlights emerging alternatives such as bacteriophage therapy, engineered probiotics, synthetic microbial communities, and CRISPR-based systems as sustainable approaches to reduce antibiotic dependence. Future research priorities include the integration of genomics, artificial intelligence, and environmental DNA monitoring for precision surveillance. This review emphasis the need for cross-sectoral collaboration, innovation, and global policy coherence to mitigate AMR in aquaculture and safeguard food, environmental, and public health security.

## 1. Introduction

The One Health framework offers an integrative lens to understand the interconnectedness of human, animal, and environmental health [1]. Its relevance to antimicrobial resistance (AMR) lies in the recognition that resistant microorganisms, antimicrobial residues, and resistance genes transcend ecological boundaries [2]. Rather than remaining confined to a single host or habitat, these elements transfer across diverse ecosystems, creating shared health challenges. Aquatic environments are central to this interconnection, functioning both as production systems for aquaculture and as conduits for microbial exchange, chemical contamination, and genetic dissemination [3].

Antimicrobial resistance has emerged as one of the most pressing global health concerns, threatening humans, animals, and the natural microbiome alike. In aquaculture systems, the selective pressure resulting from antimicrobial exposure enriches resistant bacterial populations, facilitates horizontal gene transfer, and promotes long-term persistence of resistance genes in fish, sediments, and adjacent water bodies [4]. Viewing aquaculture AMR through a One Health perspective allows for a comprehensive understanding of how resistance evolves, spreads, and affects not only aquatic organisms but also food safety, ecosystem function, and public health [5].

Aquatic ecosystems act as dynamic reservoirs and transmission hubs for resistant bacteria and genes [3]. Inputs from aquaculture, wastewater discharge, agricultural runoff, and natural microbial communities combine to create environments conducive to gene exchange among microbial species [3]. Resistance propagates through both horizontal gene transfer mechanisms and the physical movement of resistant organisms across aquatic, animal, and human populations [6]. Consequently, AMR in aquaculture must be approached not as an isolated veterinary or agricultural issue but as a complex ecological and molecular phenomenon requiring coordinated surveillance, integrated interventions, and cross-sectoral governance.

As one of the fastest-growing food-producing sectors, aquaculture plays a vital role in supporting global nutrition, employment, and economic growth. Expanding aquaculture production has become essential to meet rising protein demands and relieve pressure on capture fisheries, particularly in low- and middle-income nations [7]. Aquatic foods contribute valuable nutrients including high-quality proteins, omega-3 fatty acids, vitamins, and minerals making aquaculture indispensable to human diets and food security [8,9].

However, the intensification of aquaculture has also amplified the disease burden within farmed systems. High stocking densities, deteriorating water quality, and limited biosecurity create environments conducive to pathogen outbreaks [10]. When preventive strategies and rapid diagnostics are insufficient, the reliance on antibiotics increases, fueling resistance development among aquatic pathogens [7]. Therefore, aquaculture exists at the critical intersection of food production and AMR risk demanding innovative management systems that maintain productivity while minimizing antimicrobial use.

The implications of AMR in aquaculture extend beyond immediate farm-level challenges [11]. Resistant pathogens, antibiotic residues, and contaminated environments compromise animal welfare, consumer safety, international trade, and ecosystem resilience [12]. To safeguard food systems, it is imperative to strengthen antibiotic stewardship, enhance surveillance networks, and invest in alternative technologies and management practices [2]. Through the One Health approach, aquaculture can evolve toward a sustainable model that promotes environmental integrity, economic viability, and public health security simultaneously [3].

### 1.1. Major Pathogens in Aquaculture

Aquatic foods have gained substantial dietary importance worldwide, and the expansion of intensive culture systems has created favorable conditions for the emergence and persistence of infectious diseases. Pathogens affecting aquaculture include bacteria, parasites, fungi, and viruses, many of which are capable of causing severe mortality, production losses, and secondary complications that prompt antimicrobial intervention.

Several of these pathogens are particularly important because of their association with intensive farming systems and their relevance to animal and human health, as mentioned in Table 1. *Aeromonas* spp. are widespread in freshwater aquaculture and are notable for opportunistic behavior, environmental persistence, and the capacity to harbor resistance determinants of clinical concern. *Vibrio* spp. dominate many marine and brackish systems, while *Streptococcus iniae* is recognized not only as a fish pathogen causing severe disease and mortality but also as an emerging zoonotic organism.

Parasitic and protozoal infections further complicate disease management because they can predispose fish to bacterial coinfections and are often favored by crowding and suboptimal environmental conditions. For example, *Neoparamoeba perurans* is closely linked to amoebic gill disease in salmonids, whereas caligid copepods remain major constraints in salmon aquaculture because of tissue damage and reduced performance [13].

**Table 1:** Overview of antimicrobial resistance mechanisms in aquaculture pathogens and their relevance, including their source, hosts, and characteristics to the broader AMR framework.

SL.No.	PATHOGEN	SOURCE	HOSTS	DESCRIPTION	REFERENCES
1	*Streptococcus iniae*	Marine and freshwater environments	Tilapia (*Oreochromis niloticus*), Yellowtail (*Seriola quinqueradiata*), Rainbow trout (*Oncorhynchus mykiss*), Coho salmon (*Oncorhynchus kisutch*)	Common resistance genes include tet(M), tet(O), erm(B), erm(A), mef(A), aadA, aph(3′)-III, conferring resistance to tetracyclines, macrolides, and aminoglycosides. MDR isolates are increasingly reported in aquaculture	[14,15]
2	*Edwardsiella tarda*	Freshwater and marine aquaculture	Japanese flounder (*Paralichthys olivaceus*), Eel (*Anguilla japonica*), Tilapia (*Oreochromis niloticus*), Catfish (*Clarias* spp.)	Frequently harbors tet(A), tet(B), sul1, sul2, floR, qnrS, blaTEM, catA, leading to resistance against tetracyclines, sulfonamides, quinolones, β-lactams, chloramphenicol, and florfenicol. Plasmid mediated MDR is commonly reported	[16,17]
3	*Tenacibaculum dicentrarch*	Marine Water	European seabass (Dicentrarchus *labrax*), Atlantic salmon (*Salmo salar*), Rainbow trout (*Oncorhynchus mykiss*), Turbot (*Scophthalmus maximus*), Sole (*Solea solea*) and Gilthead sea bream (*Sparus aurata*)	Resistance has been reported against oxytetracycline, florfenicol, and sulfonamides. Resistant isolates commonly harbor tet(M), tet(B), sul1, sul2, floR, and MDR strains are increasingly detected in marine aquaculture	[18,19]
4	*Flavobacterium columnare*	Freshwater	Channel catfish (*Ictalurus punctatus*), Tilapia (*Oreochromis niloticus*), Carp (*Cyprinus carpio*), Rainbow trout (*Oncorhynchus mykiss*	Resistance has been reported to oxytetracycline and florfenicol. Resistance determinants include tet(A), tet(M), floR, with efflux pumps and biofilm formation contributing to reduced antimicrobial susceptibility. MDR isolates have emerged in intensive aquaculture	[20,21]
5	*Aeromonas* spp.	freshwater fishes	*Garra rufa*, *Catla catla*, *Labeo rohita*, *Cirrhinus mrigala*	Common ARGs include blaTEM, blaCTX-M, cphA, tetA, tetE, sul1, sul2, qnrS, floR. High prevalence of MDR strains resistant to β-lactams, tetracyclines, quinolones, sulfonamides, and phenicols.	[22,23]
6	*Mycobacterium* spp.	freshwater, marine and brackish water	striped bass (*Morone saxatilis*), Sea bass (*Dicentrarchus labrax*), *turbot* (*Scophthalmus maximus*), Florida pompano (*Trachinotus carolinus*), sygnathids	Exhibits intrinsic resistance to multiple antibiotics including rifampicin rpoB, isoniazid katG, inhA, ethambutol embB, embC, embA, and streptomycin rpsL, rrs. Biofilm formation, lipid-rich cell wall, and efflux pumps contribute to reduced antimicrobial susceptibility	[24,25]
7	*Vibrio* spp.	predominantly marine and brackish water fish, some cases of freshwater	yellow croaker (*Pseudosciaena crocea*), European sea bass (*Dicentrarchus labrax*), seahorses (*H. kuda*), Orange-spotted grouper (*Epinephelus coioides*), Tiger puffer (*T. rubripes*)	Frequently harbor tetA, tetB, sul1, sul2, qnrA, qnrS, floR, blaTEM and exhibit resistance to tetracyclines, quinolones, β-lactams, sulfonamides, and florfenicol. MDR strains are increasingly reported in aquaculture.	[26,27]

### 1.2. Antimicrobial Use (AMU) Patterns in Aquaculture

Aquaculture contributes a substantial share of global animal-derived food and continues to expand rapidly, which has increased the need for disease control under intensive production conditions [7]. In many settings, antimicrobials are used not only therapeutically but also prophylactically, and in some regions they have historically been used to compensate for inadequate husbandry, poor biosecurity, and limited access to veterinary oversight. The extent and regulation of antimicrobial use vary considerably across countries, species, and production systems, making global quantification challenging.

Antimicrobials in aquaculture are commonly administered through medicated feed and, less frequently, by immersion or bath treatment. Reported use patterns show frequent reliance on antibiotic classes that are also important in human medicine. Commonly used antibiotics include tetracyclines, sulfonamides, quinolones, and amphenicols such as florfenicol, which is widely used in aquaculture for the treatment of bacterial infections [28]. This overlap raises major One Health concerns because selective pressures in aquaculture can affect resistance to drug classes considered critically important by international health authorities.

**Figure 1:**
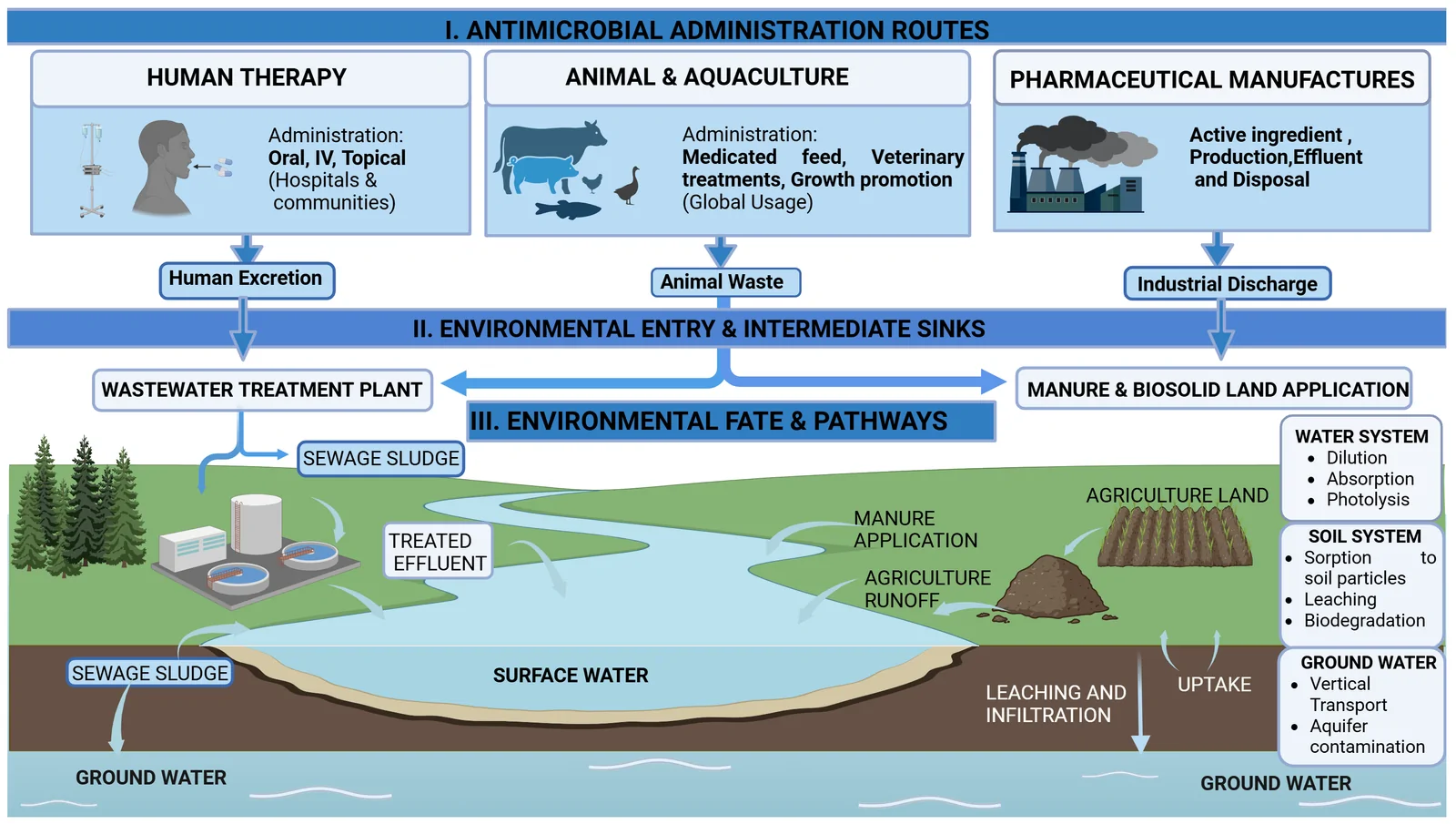
Antimicrobial administration routes and environmental fate, illustrating antimicrobial use in human therapy, animal and aquaculture production, and pharmaceutical manufacturing, followed by environmental entry through human excretion, animal waste, and industrial discharge. The schematic highlights subsequent transport, transformation, and accumulation in wastewater, surface water, soil, agricultural systems, and groundwater.

A large proportion of administered antimicrobials may be released unchanged into aquatic systems, where they persist in water or sediments and continue to exert selective pressure on microbial communities [29]. As mentioned in Figure 1, this feature distinguishes aquaculture from many terrestrial production systems, as environmental dissemination is often immediate and extensive. For that reason, antimicrobial use in aquaculture should be discussed not only in terms of drug consumption but also in relation to environmental fate, farm management practices, and the conditions that favor the emergence of resistant microbiota [5].

### 1.3. Antimicrobial Resistance in Aquaculture

**Figure 2:**
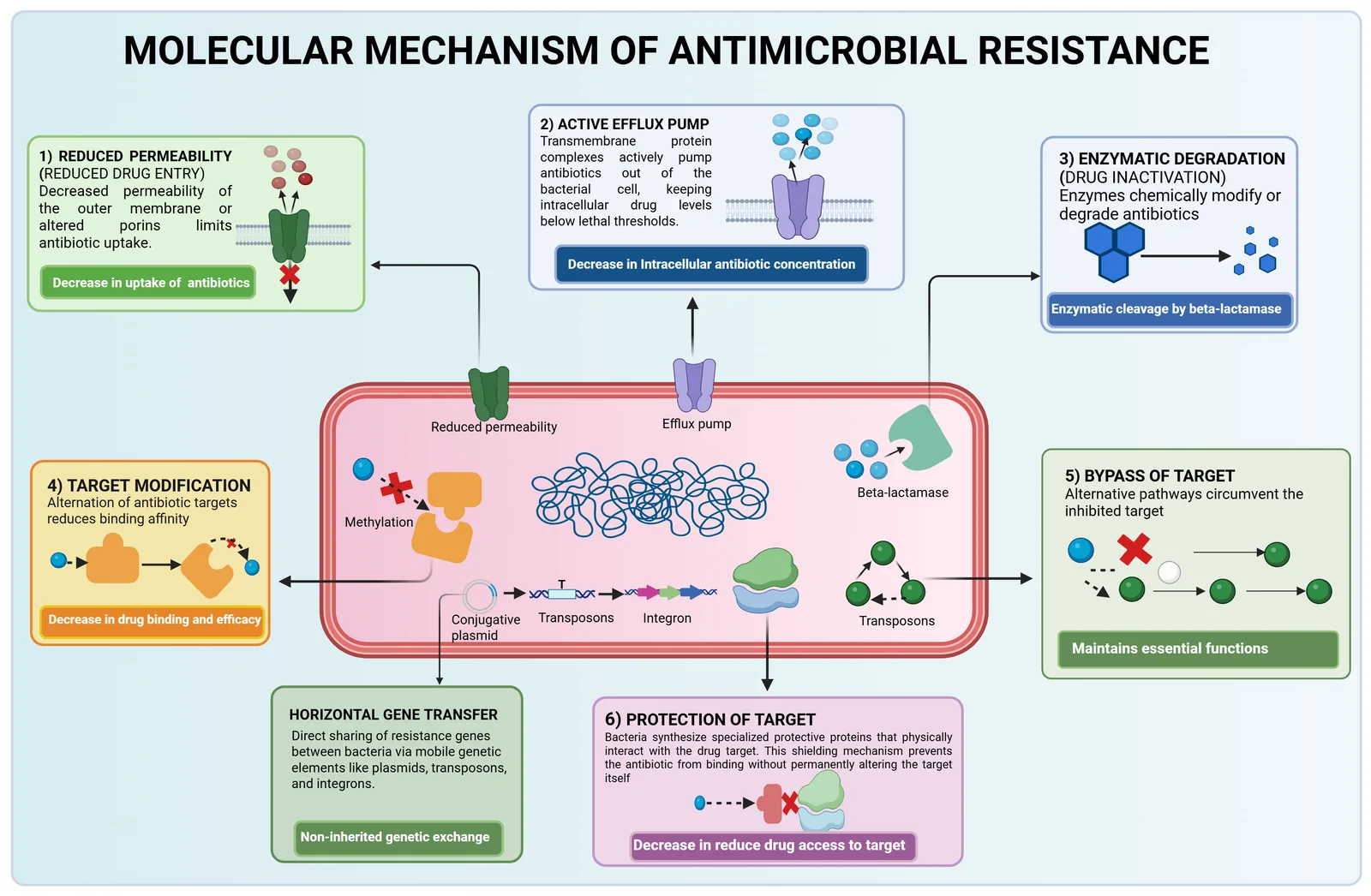
Molecular mechanisms of antimicrobial resistance, including reduced drug permeability, active efflux, enzymatic drug degradation, target modification, target bypass, target protection, and horizontal gene transfer. These mechanisms reduce antimicrobial activity and promote bacterial survival under antibiotic exposure.

The growing global population, coupled with rapid expansion of pharmaceutical industries and breeding sectors, has substantially increased antibiotic usage in aquaculture systems. This pervasive use facilitates antimicrobial resistance (AMR) development through mechanisms such as horizontal gene transfer (HGT) [30]. As mentioned in Figure 2, key resistance mechanisms include efflux pump activity, reduced cell wall permeability, enzymatic degradation of antibiotics, and modifications at drug-binding sites all of which accelerate the emergence of antibiotic-resistant bacteria (ARB) in aquatic environments [3]. Among these, plasmid-mediated resistance is particularly concerning, enabling bacteria to resist quinolone antibiotics [31]. Even after bacterial cell death, DNA persistence protected by deoxynucleotidase enzymes allows integration into other bacterial chromosomes, perpetuating resistance cycles seen in pathogens such as *Streptococcus pneumoniae* and *Vibrio cholerae* [32]. Mobile genetic elements, including integrons, conjugative plasmids, and transposons, further intensify resistance dissemination, underscoring the complex, self-propagating nature of AMR in aquaculture systems [33].

### 1.4. Prevalence and Trends of AMR in Aquaculture Settings

Aquaculture has become a crucial economic sector worldwide, offering substantial profits but also incurring serious losses due to AMR-driven infections. The misuse of antibiotics in the farming of shrimp, ornamental fish, and catfish has led to widespread resistance, with significant consequences for productivity and the global economy. Urban aquaculture environments show particularly high AMR prevalence, with dominant resistant taxa such as *Firmicutes* (18%), *Bacteroidota* (5%), *Staphylococcus* (7.3%), *Streptococcus* (2.7%), and *Corynebacterium* (1.6%).

Shrimp aquaculture across Asia, especially Malaysia, Thailand, and Vietnam, exhibit alarming levels of multidrug resistance (MDR). Malaysia reported up to 80% MDR incidence, with the first major outbreak recorded in Perak in 2010 causing severe crop failures. Common shrimp species such as *Penaeus monodon* and *Penaeus vannamei*, once highly profitable, have suffered from infection outbreaks, including Acute Hepatopancreatic Necrosis Disease caused by *Vibrio* spp. Despite efforts involving bacteriophage therapy, estimated economic losses between 2009 and 2016 reached USD 11 billion across China, Malaysia, Mexico, and Thailand [34]. In Vietnam, *Vibrio parahaemolyticus* isolates exhibit >72% resistance to tetracycline and erythromycin, with an overall prevalence of 60.25%, largely attributed to repeated water use in shrimp ponds. Similarly, the global outbreak of white spot syndrome virus in 2012 led to USD 6 billion in losses, including USD 1 billion in Asia alone [35].

Ornamental fish culture also faces substantial AMR-related losses. In Mexico, goldfish and tiger barb aquaculture generates USD 5.88 billion annually, yet AMR-associated loss is estimated to be twice that amount. Over 631 ornamental species are affected by parasitic infections associated with hemorrhagic lesions [36]. Fish to handler transmission poses an additional One Health risk. Goldfish isolates include *Pseudomonas putida*, *Comamonas aquatica*, *Aeromonas aquatica*, *A. caviae*, *A. hydrophila*, *A. sobria*, *and Edwardsiella tarda*, while tiger barb isolates commonly feature *A. hydrophila*, *A. aquatica*, *Bacillus cereus*, *Enterococcus faecalis*, *Bacillus drentensis*, *and Acinetobacter soli*, *Aeromonas aquatica* and *A. hydrophila* being predominant across species [37].

Thailand, a leading producer of finfish, demonstrates a high prevalence of resistant Gram-negative (86.6%) and Gram-positive (13.4%) bacteria. Species of *Aeromonas* and *Vibrio* spp. have caused estimated losses of USD 300 million annually in Thai aquaculture. Recent surveillance shows that Vibrionaceae (44.2%), Aeromonadaceae (32.2%), and Streptococcaceae (8.5%) isolates increased sharply between 2019 and 2021 in red tilapia and Asian seabass cultures, correlating with intensified antibiotic use [38]. Notably, AMR extends beyond aquaculture; in the U.S., methicillin-resistant *Staphylococcus aureus* causes higher mortality than HIV, Parkinson’s disease, and several other infections combined [13].

Collectively, these findings indicate that bacterial resistance in aquaculture environments has already reached nearly 80% and could approach 100% by 2050, posing a projected global economic loss of USD 60 trillion if unmitigated [39]. Addressing this crisis is vital to protect aquatic biodiversity, food security, and public health under the One Health framework [40].

### 1.5. Environmental Implications of AMR in Aquaculture

**Figure 3:**
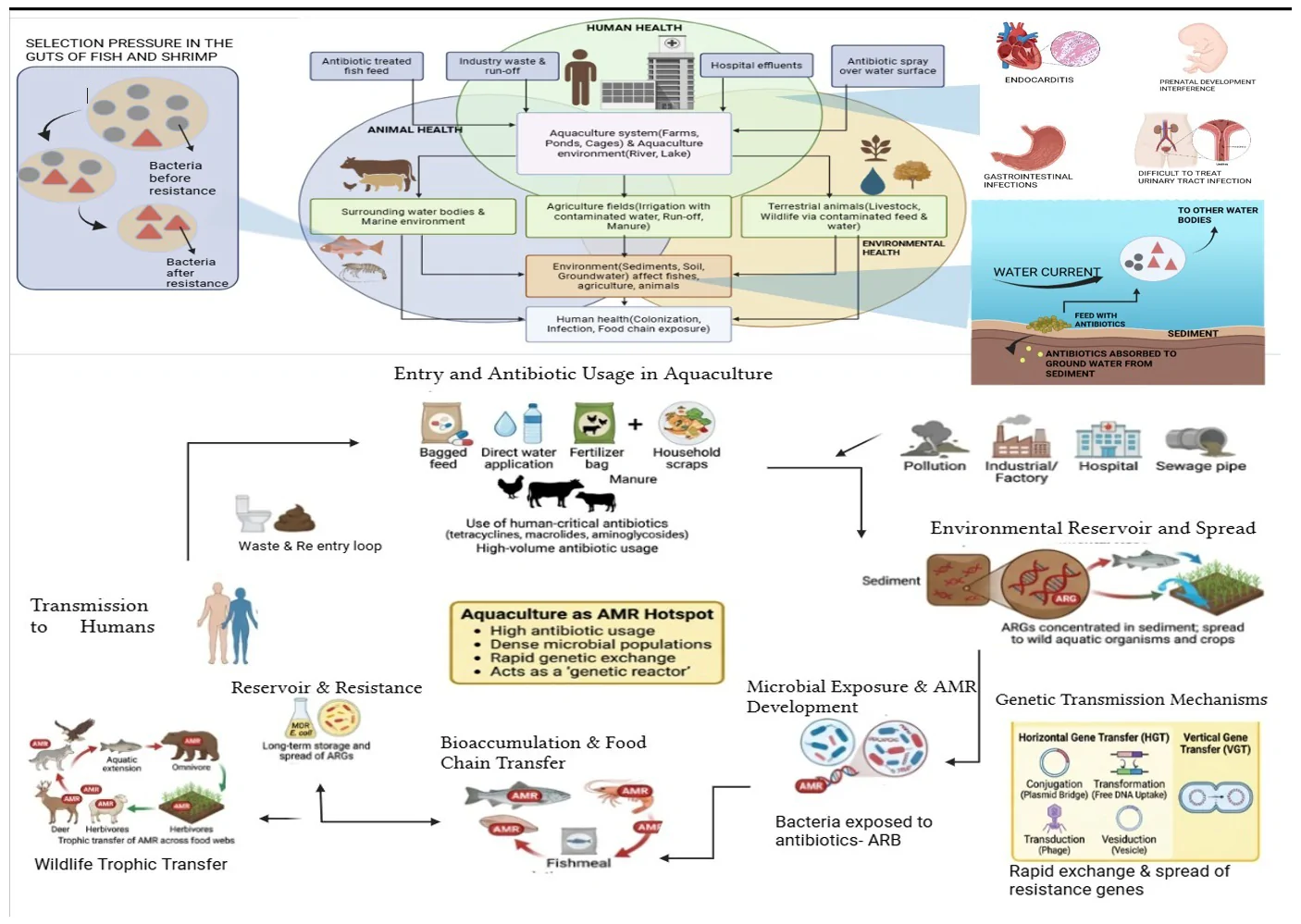
Schematic representation of how aquaculture contributes to antimicrobial resistance (AMR). The use of antibiotics in aquaculture creates selective pressure, leading to the emergence of resistant bacteria. These bacteria and resistance genes spread through water, sediments, and farmed organisms via horizontal gene transfer. Environmental pathways such as effluent discharge and runoff facilitate their movement into natural ecosystems and the food chain, linking aquatic, animal, and human health.

The environmental burden of antimicrobial resistance (AMR) arising from aquaculture is intrinsically linked to the physicochemical characteristics of antibiotics such as solubility, hydrophobicity, molecular shape, and size, which govern their interaction, absorption, and bioavailability within host organisms. Incomplete absorption or improper binding often leads to antibiotic excretion or secretion, allowing these compounds to infiltrate surrounding ecosystems through soil pores and groundwater, thereby facilitating the dissemination of antibiotic resistance genes (ARGs) across environmental matrices as shown in Figure 3. It is estimated that nearly 75% of the antibiotics administered in aquaculture are delivered via feed, of which a considerable fraction remains unmetabolized. These residues escape into the water column or sediments, driving chemical transformation and ecological disruption. For instance, oxolinic acid, a quinolone derivative, has been shown to impair the reproductive capacity of aquatic invertebrates such as *Daphnia* spp. [12].

The ecological ramifications extend throughout the aquatic food web. Antibiotic residues exert toxic and sub-lethal effects on phytoplankton, zooplankton, and microalgae organisms foundational to aquatic productivity and nutrient cycling. Such disruptions impede photosynthetic efficiency, alter primary productivity, and potentially lead to oxygen depletion. Since algae serve as oxygenic autotrophs essential to both aquatic and atmospheric oxygen balance, antibiotic-induced stress on algal populations can cascade into broad ecological instability, ultimately affecting the survival and reproductive success of higher trophic organisms [36].

Human exposure to ARB derived from aquaculture can occur through direct contact, human-to-human transmission, handling of pets, or consumption of raw or undercooked aquatic products. Despite their critical implications, the transmission and dissemination of AMR from environmental reservoirs and aquaculture operations have received comparatively less attention than those occurring within clinical or terrestrial animal settings [3]. Wastewater treatment plants, hospital effluents, and agricultural runoff act as hotspots for ARG persistence and gene exchange, contributing to the emergence of novel resistant variants later reintroduced into natural ecosystems [5].

The use of antibiotic-supplemented animal feeds to promote growth and prevent disease also contributes indirectly through manure application in agriculture. These manures, upon leaching, contaminate surface and groundwater sources and further amplify horizontal gene transfer through mechanisms such as transformation, transduction, and conjugation [3,41]. Environmental persistence and transfer dynamics are further influenced by climatic factors, with AMR propagation documented across temperature extremes from cold aquatic systems to geothermal hot springs.

Wildlife species are not exempt from this cycle. As resistance circulates through food webs, antibiotic exposure in wild animals often results in therapeutic inefficacy due to pre-existing mutations, prompting higher dosage use and consequently escalating ARB proliferation. Recognizing the escalating environmental threat, India introduced the Environment (Protection) Amendment Rules in 2019 to regulate pharmaceutical emissions and mitigate AMR spread [5]. Nevertheless, without integrated interventions under a One Health framework, as mentioned in Figure 3, environmental AMR may soon become a defining global challenge, endangering ecosystem stability, food security, and human health [42].

## 2. Human Health Implications

In aquaculture, antibiotics are routinely incorporated into feed to prevent and treat bacterial infections in farmed fish. However, uneaten feed and residual antibiotics often accumulate in sediments and are transported over long distances by water currents. These residues serve as reservoirs for antibiotic resistance genes (ARGs), which can be transferred to human pathogens through horizontal gene transfer (HGT) mechanisms such as conjugation [43]. During conjugation, ARGs move from donor to recipient cells via direct physical contact, frequently mediated by mobile genetic elements such as plasmids that carry multiple resistance determinants [32]. For example, *Aeromonas species* common fish pathogens share ARGs with *Escherichia coli* through IncU plasmids harboring tetracycline resistance genes, enabling exchange between *Aeromonas salmonicida*, *A. hydrophila*, *A. caviae*, and human-associated *E. coli* [44].

Beyond aquatic microbial transfer, humans face direct exposure to resistant bacteria through seafood consumption. The risk escalates when aquatic products are eaten raw or undercooked, such as sushi or cold-smoked salmon [45]. Consumption of shellfish like oysters and shrimp contaminated with *Aeromonas hydrophila* can lead to gastrointestinal infections, while biofilm-forming multidrug-resistant *Enterococcus faecalis* in seafood complicates treatment of bacteremia, urinary tract infections, and endocarditis in humans [3,36]. Adverse reactions to antibiotic residues in aquatic food such as urticaria and angioneurotic edema, have also been documented. Chronic exposure to low levels of antibiotic residues may result in organ lesions due to prolonged tissue accumulation. Quinolones and tetracyclines, two widely used antibiotic classes in aquaculture, can interfere with tooth development in children, whereas erythromycin and furazolidone residues have been linked to deafness, peripheral neuritis, hemolytic anemia, and polyneuritis.

Among resistant pathogens, extended-spectrum β-lactamase (ESBL)-producing bacteria pose significant concern. The presence of ESBL-producing *E. coli* in seafood represents a major public health threat, as these strains can spread through the food chain and cause complicated infections such as urinary tract infections and septicemia [46]. Quinolones, classified as Veterinary Critically Important Antimicrobials (VCIAs), account for approximately 27% of antibiotics used in aquaculture, yet fluoroquinolone exposure has been associated with nearly 70% of AMR-related deaths worldwide [12]. Transmission can also occur through contaminated drinking water, where resistant aquaculture pathogens infiltrate groundwater systems [47].

In addition to microbial hazards, chemical contaminants from aquaculture exert extensive health impacts. Farmed fish may accumulate methylmercury (MeHg), persistent organic pollutants (POPs), and veterinary drugs through feed and fish oils derived from wild-caught species such as herring and sardines [39]. MeHg bioaccumulates through the aquatic food web and constitutes a major route of human exposure. Prenatal MeHg exposure can cross the placenta and blood–brain barrier, impairing neurodevelopment and reducing intelligence quotient (IQ), while adult exposure increases cardiovascular disease risk. Further, the widespread use of algaecides, herbicides, and pesticides in aquaculture for disease prevention contributes to chronic toxicity and heightened incidence of non-communicable diseases, including cancer. Diflubenzuron, an insecticide commonly applied in aquaculture, persists in marine sediments for up to seven months, compounding long-term ecological and health concerns. Chloramphenicol residues in animal-derived foods are associated with irreversible and often fatal aplastic anemia.

To address these safety concerns, global regulatory frameworks have been established. The Food and Agriculture Organization (FAO) and World Health Organization (WHO), through the Codex Alimentarius Commission, have defined residue monitoring standards and established Acceptable Daily Intake (ADI) values the estimated level of residue that can be safely consumed daily over a lifetime without health risk. Collectively, these findings highlight that the aquatic environment functions as a critical conduit linking antimicrobial use in aquaculture with the emergence of antimicrobial resistance in humans [48]. This underscores the necessity of adopting the One Health approach to curtail antibiotic misuse and safeguard human health on a global scale.

## 3. Animal Health and Welfare

### 3.1. Impact of AMR on Aquaculture Animal Health

Climate change has emerged as a critical global challenge with profound consequences for aquatic ecosystems and aquaculture health. Rising water temperatures, fluctuations in precipitation patterns, and an increased frequency of extreme climatic events collectively influence water quality, pathogen distribution, virulence, and host susceptibility.

Moreover, heightened atmospheric carbon dioxide levels accelerate ocean acidification and disrupt pH balance, compromising physiological homeostasis in fish and increasing vulnerability to infectious diseases [49]. Concurrently, the intensive and often unregulated use of antibiotics in aquaculture has accelerated the selection and dissemination of antimicrobial resistance (AMR). The combination of high stocking densities, subtherapeutic feed medication, and prophylactic antibiotic applications fosters selective pressure that promotes the emergence of multidrug-resistant (MDR) bacterial strains. These MDR pathogens not only threaten aquatic animal health but also pose major food safety and global health risks by facilitating resistance gene transfer through the food chain [50].

Among aquatic bacterial pathogens, *Escherichia coli* represents a major challenge, contributing to both economic losses and zoonotic threats. Pathogenic *E. coli* strains induce lesions, hemorrhages, and impaired growth performance in fish, with infection dynamics amplified under elevated temperatures and changing water conditions that favor bacterial proliferation [51]. The organism typically enters the host through contaminated water or feed, forms biofilms on surface tissues, and secretes toxins that cause extensive intestinal and systemic pathology. Likewise, *Streptococcus* species such as *S. agalactiae* and *S. iniae* are among the most significant aquaculture pathogens, causing high mortality, neuroinvasive symptoms, and septicemia that severely affect production. Additional pathogens, including *Flavobacterium columnare* and *Francisella* spp., have also been frequently reported, exacerbating disease burdens across aquaculture systems [52].

Many fish-associated bacteria have zoonotic potential, making aquaculture an important interface in the One Health continuum. The microbial flora of fish reflects that of their aquatic environment and fluctuates with changes in water quality, nutrient load, and temperature [53]. Contamination from agricultural runoff or sewage discharge can compromise inshore and filter-feeding species, particularly mollusks, leading to colonization by pathogens such as *Salmonella typhi*, *Vibrio* spp., and *Campylobacter* spp. While consumption of raw or undercooked seafood remains a considerable food safety risk, proper cooking significantly mitigates the likelihood of infection transmission [54].

### 3.2. Strategies for Improving Animal Welfare and Reducing AMR

The sustainability of aquaculture depends not only on productivity but also on prudent antimicrobial stewardship and welfare-centered management practices. Incorporating probiotics has emerged as a promising and eco-friendly strategy to enhance fish health while reducing antibiotic reliance. Probiotics, commensal and beneficial microbes including bacteria, yeasts, fungi, and bacteriophages, can modulate gut microbiota, stimulate immune responses, inhibit pathogen colonization, and promote overall health and growth performance in cultured species [55].

### 3.3. Global Surveillance and Stewardship Initiatives

Standardized global surveillance of antimicrobial consumption (AMC) in animals is vital to manage resistance emergence [56]. The World Organisation for Animal Health (WOAH) established the ANIMUSE global database to compile country-specific AMR and AMC data, expressed as milligrams of antimicrobial per kilogram of estimated animal biomass [56]. In 2023, 157 countries participated, though data transparency remains limited since public country-level reporting is voluntary. Harmonized data collection enables cross-country comparisons and supports evidence-based policymaking [56]. However, several challenges persist weak regulatory systems, inconsistent data quality, limited digital infrastructure, and economic resistance from key stakeholders such as farmers and pharmaceutical industries [57]. While ANIMUSE has strengthened global coordination of monitoring systems, national-level policy enforcement and transparency continue to lag behind.

## 4. Public–Private Collaboration in Aquaculture Production

Strengthening public–private partnerships within aquaculture and livestock production systems serves as a key driver in combating AMR. Collaborative governance models that integrate government oversight with industry engagement encourage responsible antibiotic use without compromising productivity. A successful example can be drawn from Denmark’s pig and aquaculture management framework, where integrated cooperation among authorities, veterinarians, producers, and farmer associations has dramatically reduced antimicrobial use [57]. The “Yellow Card” scheme an antibiotic threshold-based monitoring system sets consumption limits at the farm level, triggering regulatory action when thresholds are exceeded. This initiative, supported by investment in biosecurity, herd management, and animal welfare measures, has proven effective in lowering AMR while maintaining profitability. Such incentive-driven, transparent, and data-informed strategies demonstrate that coordinated control mechanisms can enhance both animal health and public trust, ensuring sustainable aquaculture systems aligned with One Health principles [57].

Collectively, integrating responsible antibiotic stewardship, biological alternatives like probiotics, and collaborative policy frameworks represents a scalable approach to minimize AMR in aquaculture while safeguarding animal welfare, food safety, and global health security.

### 4.1. Integrated Surveillance Strategies for Addressing AMR in Aquaculture

The future control of antimicrobial resistance (AMR) depends on effective collaboration across all sectors of human health, animal health, and environmental management guided by the One Health (OH) principle. Strengthening antimicrobial stewardship and implementing robust surveillance systems that span wastewater, farms, and natural ecosystems are essential. Wastewater surveillance exemplifies the One Health approach by providing a scalable, real-time framework for tracking AMR trends and identifying hotspots across interconnected systems. Marine sediments, often acting as reservoirs of resistant bacteria and antibiotic residues, reflect downstream accumulation caused by aquaculture discharge, municipal effluents, and industrial runoff. Advanced wastewater treatment technologies such as ozonation, ultraviolet (UV) disinfection, and membrane filtration can substantially mitigate the release of antibiotics and resistance genes into aquatic environments, offering critical protection for farmed species and surrounding ecosystems [58].

The intrinsic interconnection between humans, animals, and their environment underscores the necessity of adopting integrated policies that couple antimicrobial stewardship with coordinated surveillance and international cooperation. Addressing AMR in aquaculture requires systematic monitoring of environmental contamination, improved husbandry practices, and judicious antimicrobial use. Environmental surveillance, particularly in coastal zones and aquaculture systems, plays a pivotal role in early detection and risk assessment of emerging resistance patterns [59]. Employing molecular tools such as metagenomic sequencing in water and sediment sampling provides detailed insights into resistance gene prevalence and dissemination dynamics. Global initiatives including the WHO Global Action Plan on AMR and partnerships with the FAO and WOAH exemplify the need for global multisectoral collaboration to reduce AMR transmission from aquaculture to human populations [46].

A comprehensive One Health research framework must integrate human, animal, environmental, and wildlife health perspectives to drive actionable, cross-sector interventions. Stakeholder engagement and knowledge exchange among public health, veterinary, environmental, and wildlife sectors enhance the prevention of resistance emergence and spread. Quadripartite Collaboration comprises the Food and Agriculture Organization (FAO), World Health Organization (WHO), World Organisation for Animal Health (WOAH), and United Nations Environment Programme (UNEP). Incorporating environmental and wildlife data into national and global AMR action plans further strengthens surveillance capacity and the overall impact of mitigation measures [22].

The One Health concept highlights the interdependence of environmental, animal, and human ecosystems, a relationship especially critical to addressing AMR within aquaculture. One Health interventions encompass preventive and alternative management strategies, including the use of prebiotics, probiotics, vaccines, peptides, and bacteriophage therapy, along with rigorous biosecurity, surveillance, and education programs aimed at both producers and consumers. Reducing antimicrobial use (AMU) while enhancing surveillance at farming (pre-harvest) and processing (post-harvest) stages is central to risk mitigation. Integrating environmental data such as water quality and AMR gene detection into monitoring systems ensures informed, evidence-based decision-making. Strengthened cross-sectoral coordination between human, animal, and environmental agencies, alongside enforcement through frameworks led by the EMA, WHO, and WOAH, remains vital, particularly in low- and middle-income countries where environmental integration often lags [25,60].

Regional programs exemplify these principles. The Southeast Asian One Health Universities Network focuses on workforce development, education, and practical training to address interrelated health risks, including aquaculture driven AMR. Surveillance systems, such as the Acute Respiratory Infection Surveillance Program and biosurveillance networks, extend One Health methodologies through timely data sharing and outbreak alerts. Initiatives like Indonesia’s Sistem Informasi Zoonosis dan Emerging Infectious Diseases and Singapore’s integrated hospital surveillance programs demonstrate how multisectoral information platforms enhance preparedness and can be adapted for aquaculture-associated surveillance. Likewise, Thailand’s “raised-without-antibiotics” standards in aquaculture and livestock showcase successful policy industry collaboration in reducing antibiotic consumption. The integration of advanced data analytics including artificial intelligence and machine learning can further optimize surveillance, allowing predictive modeling and adaptive risk management for AMR mitigation [25,61].

The FAO, WOAH, WHO, and United Nations Environment Programme (UNEP) jointly advocate the One Health approach as a unified strategy for monitoring antimicrobial usage and resistance globally. Norway was a pioneering example, establishing surveillance across veterinary and human sectors in the late 1990s, yet systematic evaluations of the “One Healthness” of its AMR systems remain pending. In Europe, the Evaluation Tool for One Health Epidemiological Surveillance Capacities and Capabilities (OH-EpiCap), developed through the One Health European Joint Programme, enables countries to assess intersectoral cooperation in pathogen monitoring and quantify the integration level of their OH surveillance systems. Similar initiatives, such as Canada’s One Health Evaluation Antimicrobial Use and Resistance Surveillance (OHE-AMURS), assess how coordination across federal, provincial, and territorial levels strengthens stewardship and data-sharing frameworks. Canada’s model highlights the transition from pilot projects to institutionalized, well-resourced national surveillance with sustainable governance, inclusivity, and resilience [62].

Other national programs echo these trends. Thailand’s National Strategic Plan on Antimicrobial Resistance (2017–2021) proposed incorporating antimicrobial residue and consumption data into its national surveillance platform under the One Health framework. Parallel progress in Sri Lanka illustrates regional adaptation where studies examining AMR patterns in E. coli across human, poultry, and aquaculture sectors inform groundwork for a unified surveillance system integrating laboratory testing, public health research, and data harmonization. Such country-level efforts show tangible progress toward structured One Health implementation through consistent, evidence-driven planning [63].

Genomic surveillance further transforms resistance monitoring by enabling high-resolution tracing of AMR genes, their origins, persistence, and transfer routes. Standardized protocols connecting genomic data with phenotypic and environmental datasets enhance cross-sector interpretation and intervention design. Interdisciplinary genomic research initiatives that bridge human, animal, plant, and environmental health strengthen our ability to track resistance pathways and guide source attribution [64].

Sustainable AMR mitigation also depends on collaboration at both governance and operational levels. Governance-level cooperation involves cross-sectoral working groups, scientific committees, and policy institutions that coordinate strategic direction, capacity-building, and regulatory implementation. Operational-level collaboration, in contrast, emphasizes joint methods for data collection, analysis, and information dissemination to guide responsive management. Integrated surveillance defined as systemic, cross-sectoral monitoring of antimicrobial use and resistance to inform mitigation decisions remains the cornerstone of effective One Health governance. It promotes co-created knowledge, aligns monitoring with stewardship, and ensures that policies are informed by continuous assessment. Strengthening integrated AMR surveillance within aquaculture environments will therefore remain central to safeguarding ecosystem health, food safety, and global well-being [25].

### 4.2. Policy and Regulations on Antimicrobial Use in Aquaculture

Antimicrobial resistance (AMR) represents one of the most pressing global health challenges, arising when microorganisms acquire mechanisms to withstand therapeutic interventions. In aquaculture, the widespread misuse and overuse of antibiotics for disease prevention or accelerated growth has catalyzed the emergence of resistant bacterial strains such as *Escherichia coli* and *Vibrio* spp. These resistant microbes, once established in aquatic environments, have the capacity to persist, spread through water systems, and enter the human food chain, posing significant threats to food safety and public health. This escalating concern demands regulatory scrutiny under a unified global framework [65].

International organizations including the World Health Organization (WHO) and Food and Agriculture Organization (FAO) have advanced comprehensive policy initiatives grounded in the One Health approach, emphasizing the interdependence of human, animal, and environmental health. These frameworks guide national and regional policy development, encouraging responsible antibiotic stewardship, reduced non-therapeutic use, and sustainable aquaculture practices. The One Health perspective ensures that interventions are neither isolated nor sector-specific but instead integrate across health systems to collectively combat AMR proliferation [25].

Presently, regulatory governance for antimicrobial use in aquaculture primarily focuses on ensuring judicious application, monitoring, and enforcement. Many countries have restricted or banned antibiotics for growth promotion, authorizing therapeutic use only under veterinary supervision. Agencies such as the U.S. Food and Drug Administration (FDA) and equivalent national authorities provide detailed guidance on dosage, withdrawal periods, and approved antimicrobial classes for aquaculture. Concurrently, surveillance protocols assess antimicrobial residues, monitor resistant bacterial strains, and examine seafood safety before products reach markets [7].

Despite these efforts, major challenges persist, particularly the widespread availability of antibiotics without prescription and the limited awareness of responsible antimicrobial use among aquaculture practitioners. In many regions, weak regulatory enforcement facilitates the over-the-counter sale and indiscriminate use of antibiotics, leading to the accumulation of antibiotic residues in aquatic ecosystems and the expansion of antimicrobial resistance reservoirs. Inadequate waste management practices and ineffective water governance further exacerbate these impacts by promoting the persistence and dissemination of antibiotic resistance genes (ARGs) within natural microbial communities [25].

Addressing these policy and implementation gaps requires a comprehensive and coordinated approach. First, regulatory systems should be fortified with stronger compliance mechanisms and international harmonization of surveillance standards. Second, farmer education and public awareness programs must translate scientific knowledge into practical management behaviors that curb misuse. Finally, future policy directions should prioritize a shift away from antibiotic reliance by promoting preventive strategies such as vaccines, probiotics, immunostimulants, and robust biosecurity protocols that sustain productivity while protecting ecosystem integrity [8].

In this context, the evolution of aquaculture policy must balance productivity demands with ecological and public health safeguards. Developing regulatory frameworks that not only limit antibiotic use but also encourage innovation in sustainable aquaculture technologies represents the most viable path forward. The One Health-guided integration of policy, education, and scientific advancement offers a holistic blueprint for addressing AMR at its aquatic origin, ultimately ensuring safer food systems and healthier global communities [66].

## 5. Future Directions and Research Needs

As aquaculture continues to expand globally as a vital source of nutrition and economic growth, antibiotic dependency remains a persistent challenge in disease management. The overreliance on conventional antibiotics for controlling bacterial infections not only drives antimicrobial resistance (AMR) but also undermines ecosystem sustainability and market confidence [65]. Future research must therefore focus on innovative strategies that integrate biological, technological, and environmental interventions to reduce antibiotic use while ensuring aquatic animal health [67,68].

### 5.1. Novel Therapeutic Approaches: Bacteriophages and SynComs

One of the most promising alternatives to antibiotic therapy is the use of bacteriophages viruses that selectively infect and destroy bacterial pathogens. Phage therapy offers high specificity, minimal ecological disturbance, and the potential for co-evolution with target microorganisms. Current research prioritizes the formulation of phage cocktails, combinations of lytic phages designed to minimize the risk of resistance development by targeting multiple bacterial strains simultaneously. Polyvalent phages, capable of attacking different strains within the same species, further enhance therapeutic effectiveness and adaptability in diverse aquaculture systems [69].

**Figure 4:**
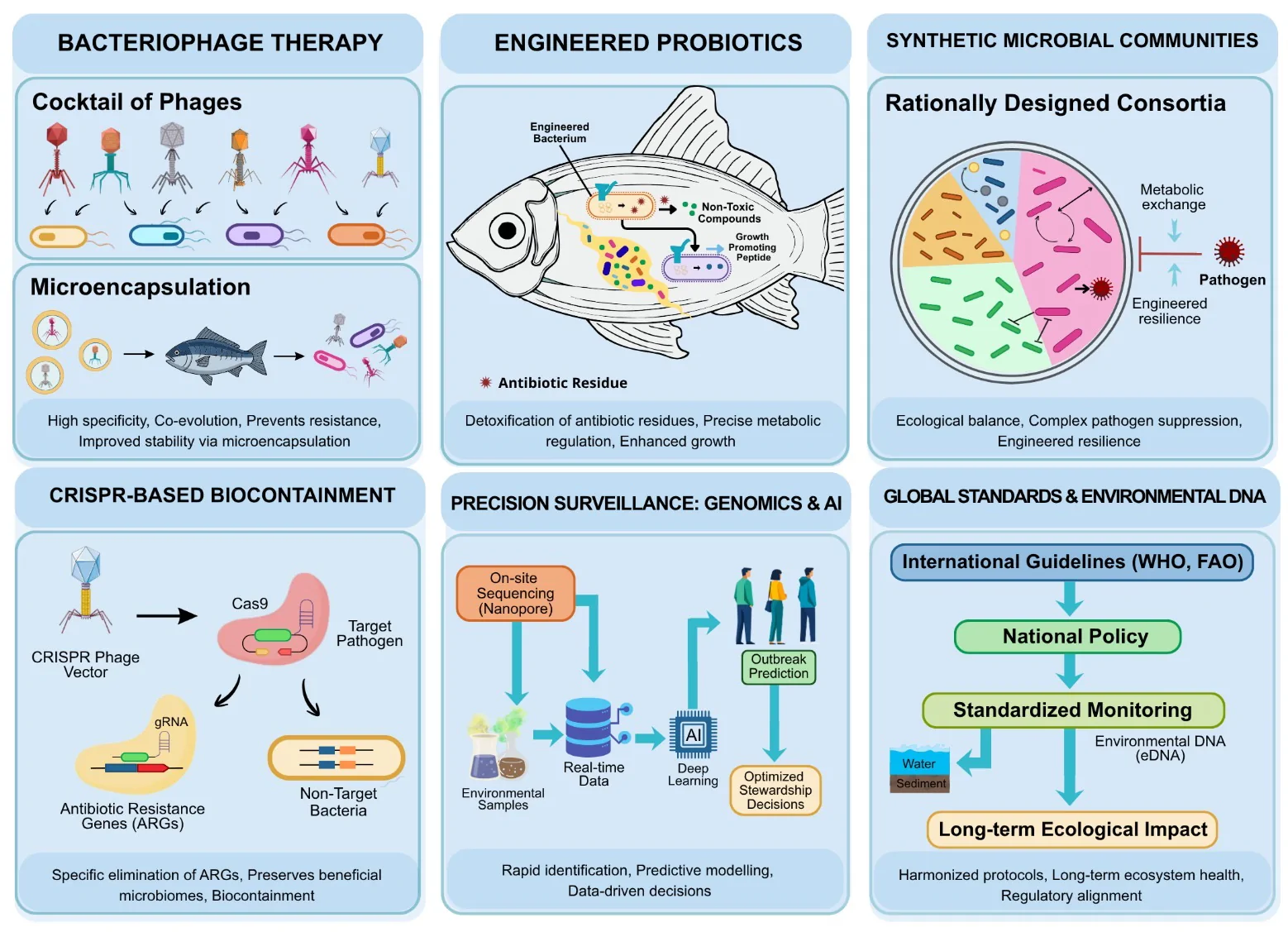
Emerging strategies for antimicrobial resistance management in aquaculture, including phage therapy, engineered probiotics, synthetic microbial communities, CRISPR-based biocontainment, and precision surveillance. The integration of genomics, AI, eDNA monitoring, and global standards supports targeted pathogen control, early AMR detection, and sustainable aquaculture management.

Advances such as microencapsulation have improved the practical stability of phage applications by providing protective coatings that allow phages to survive unfavorable conditions and deliver targeted activity upon encountering host bacteria. In Figure 4 it is demonstrated that the integration of bacteriophage therapy into aquaculture disease management represents a transformative direction, yet global standardization of protocols especially in low income regions remains limited and urgently needed to ensure safety, efficacy, and reproducibility across ecological contexts.

### 5.2. Integrating Genomic and AI Technologies

Emerging molecular and computational techniques have revolutionized our ability to track, predict, and manage AMR in aquaculture. Nanopore sequencing facilitates rapid, real-time DNA sequencing without dependence on sophisticated laboratory infrastructure, enabling onsite pathogen identification and resistance profiling. Artificial intelligence (AI) complements these workflows by analyzing complex datasets to predict outbreak risks and optimize intervention strategies. Likewise, single-cell genomics allows researchers to observe bacterial responses to treatments at a cellular level, providing critical insights into adaptation mechanisms.

Metagenomics is particularly valuable in identifying naturally occurring bacteriophages capable of lysing resistant pathogens such as *Aeromonas hydrophila* and *Vibrio parahaemolyticus*. Whole-genome sequencing of these phages enables in-depth safety evaluation by confirming the absence of virulence or resistance genes. Collectively, these genomic tools contribute to precision aquaculture, allowing data-driven decisions about microbial management and drug stewardship.

Advances in synthetic biology are opening new frontiers for combating AMR in aquaculture. Engineered probiotic strains are being developed to detect antibiotic residues and metabolize them into non-toxic compounds, thus reducing chemical contamination and secondary resistance emergence. Similarly, CRISPR-based biocontainment strategies leverage modified bacteriophages to selectively eliminate ARGs (antibiotic resistance genes) from bacterial populations while preserving non-pathogenic microbes. Rationally designed synthetic microbial communities (SynComs), composed of synergistic natural microorganisms, represent another frontier for maintaining ecological balance and suppressing pathogenic growth. While these engineered systems remain in experimental phases and face stability limitations in open aquatic environments, understanding phagehost–microbiome dynamics will be key to overcoming these barriers [65,70].

### 5.3. Research Priorities and Implementation Challenges

Despite advances, key research gaps persist in aquaculture AMR management. Future directions should emphasize:

1. Development of host-specific bacteriophages and standardized therapeutic protocols for regulatory adoption.
2. Integration of multi-omics approaches genomics, transcriptomics, proteomics, and metabolomics into probiotic design and environmental risk assessment.
3. Implementation of environmental DNA (eDNA) monitoring systems to evaluate the long-term ecological effects of antimicrobial interventions.
4. Establishment of international phage therapy guidelines that harmonize efficacy, safety, and biocontainment strategies across aquaculture sectors.

Authoritatively, the next research horizon must embrace a transdisciplinary model, engaging microbiologists, aquaculture experts, data scientists, and environmental policymakers to devise holistic AMR mitigation strategies. Collaborative research hubs and shared genomic repositories should facilitate real-time data exchange, bridging the scientific–policy gap that often limits effective AMR oversight.

### 5.4. Aligning with Global Sustainable Goals

The future of AMR research in aquaculture must align with the United Nations Sustainable Development Goals (SDGs), particularly SDG 13: Climate Action and SDG 14: Life Below Water. These global objectives underscore the imperative of ecosystem restoration, responsible antimicrobial use, and sustainable food production. Strong coordination among researchers, government authorities, and industry stakeholders will be indispensable for implementing environmentally sound and socially equitable aquaculture practices. From the authors’ perspective, future success lies in shifting the paradigm: moving from reactive antibiotic management to proactive microbial ecology and precision biotherapeutics. Integrated multidisciplinary frameworks—supported by cutting-edge genomics and strong international collaboration—can transform aquaculture into a model system for One Health-driven AMR mitigation. Investing in these directions today is critical to securing aquatic biodiversity, food safety, and global health security for generations ahead.

## 6. Conclusions

The challenge of antimicrobial resistance (AMR) in aquaculture exemplifies a global health issue that transcends disciplinary and geographic boundaries. From molecular mechanisms driving resistance evolution to environmental dissemination and public health consequences, AMR represents an interconnected cycle sustained by current production practices, insufficient regulation, and environmental contamination. Addressing this complex problem requires reframing aquaculture not merely as a food production system but as a dynamic component of a broader ecological and health network.

A One Health perspective offers the most comprehensive pathway forward—integrating human, animal, and environmental health into a unified strategy. Strengthened antimicrobial stewardship, standardized surveillance, and regulatory enforcement must form the backbone of AMR mitigation globally. Concurrently, environmental monitoring and wastewater management are essential to restrict the spread of resistance genes within aquatic ecosystems. Sustainable disease management should shift from reliance on antibiotics to the adoption of biological innovations such as bacteriophages, engineered probiotics, vaccines, and synthetic microbial consortia, supported by genomic, metagenomic, and AI-driven monitoring technologies.

Future aquaculture systems must move toward precision, prevention, and partnership—precision through data-driven decision-making, prevention via biosecurity and ecological resilience, and partnership through collaboration among researchers, policymakers, and industry stakeholders. Aligning these actions with international frameworks and the UN Sustainable Development Goals (particularly SDG 13 and SDG 14) will be critical to foster sustainable aquaculture that safeguards food security, protects biodiversity, and promotes global health equity. The success of AMR mitigation in aquaculture will ultimately depend on our collective capacity to transform scientific understanding into coordinated, accountable, and enduring One Health action.

## Data Availability

The original contributions presented in this study are included in the article. Further inquiries can be directed to the corresponding authors.

## Abbreviations

The following abbreviations are used in this manuscript:

AMR	Antimicrobial Resistance
UV	Ultraviolet
Spp.	Species
HGT	Horizontal Gene Transfer
MDR	Multidrug Resistance
ARGs	Antibiotic Resistance Genes
*A. hydrophila*	*Aeromonas hydrophila*
VCIAs	Veterinary Critically Important Antimicrobials
POPs	Persistent Organic Pollutants
FAO	Food and Agriculture Organization
ADI	Acceptable Daily Intake
WOAH	World Organisation for Animal Health
AMC	Antimicrobial Consumption
UNEP	United Nations Environment Programme
FDA	Food and Drug Administration
ESBL	Extended-Spectrum β-lactamase
eDNA	environmental DNA
CRISPR	Clustered Regularly Interspaced Short Palindromic Repeats
WHO	The World Health Organization

## References

[1] Al-Khalaifah H., Rahman M.H., Al-Surrayai T., Al-Dhumair A., Al-Hasan M. (2025). A One-Health Perspective of Antimicrobial Resistance (AMR): Human, Animals and Environmental Health. Life.

[2] McEwen S.A., Collignon P.J. (2018). Antimicrobial Resistance: A One Health Perspective. Microbiol. Spectr..

[3] Cabello F.C., Godfrey H.P., Buschmann A.H., Dölz H.J. (2016). Aquaculture as yet Another Environmental Gateway to the Development and Globalisation of Antimicrobial Resistance. Lancet Infect. Dis..

[4] Reverter M., Sarter S., Caruso D., Avarre J.-C., Combe M., Pepey E., Pouyaud L., Vega-Heredía S., de Verdal H., Gozlan R.E. (2020). Aquaculture at the Crossroads of Global Warming and Antimicrobial Resistance. Nat. Commun..

[5] Watts J.E.M., Schreier H.J., Lanska L., Hale M.S. (2017). The Rising Tide of Antimicrobial Resistance in Aquaculture: Sources, Sinks and Solutions. Mar. Drugs.

[6] Larsson D.G.J., Flach C.-F. (2022). Antibiotic Resistance in the Environment. Nat. Rev. Microbiol..

[7] Rizzo L., Manaia C., Merlin C., Schwartz T., Dagot C., Ploy M.C., Michael I., Fatta-Kassinos D. (2013). Urban Wastewater Treatment Plants as Hotspots for Antibiotic Resistant Bacteria and Genes Spread into the Environment: A Review. Sci. Total Environ..

[8] Schar D., Klein E.Y., Laxminarayan R., Gilbert M., Van Boeckel T.P. (2020). Global Trends in Antimicrobial Use in Aquaculture. Sci. Rep..

[9] Hicks C.C., Cohen P.J., Graham N.A.J., Nash K.L., Allison E.H., D’Lima C., Mills D.J., Roscher M., Thilsted S.H., Thorne-Lyman A.L. (2019). Harnessing Global Fisheries to Tackle Micronutrient Deficiencies. Nature.

[10] Golden C.D., Koehn J.Z., Shepon A., Passarelli S., Free C.M., Viana D.F., Matthey H., Eurich J.G., Gephart J.A., Fluet-Chouinard E. (2021). Aquatic Foods to Nourish Nations. Nature.

[11] Murray A.G., Peeler E.J. (2005). A Framework for Understanding the Potential for Emerging Diseases in Aquaculture. Prev. Vet. Med..

[12] Antimicrobial Resistance Collaborators (2022). Global Burden of Bacterial Antimicrobial Resistance in 2019: A Systematic Analysis. Lancet.

[13] Schar D., Zhao C., Wang Y., Larsson D.G.J., Gilbert M., Van Boeckel T.P. (2021). Twenty-Year Trends in Antimicrobial Resistance from Aquaculture and Fisheries in Asia. Nat. Commun..

[14] Vallarino M.C., Dagen S.L., Costelloe E., Oyenekan S.I., Tinsley J., Valdenegro V., Król E., Noguera P., Martin S.A.M. (2024). Dynamics of Gill Responses to a Natural Infection with *Neoparamoeba perurans* in Farmed Tasmanian Atlantic Salmon. Animals.

[15] Rigos G., Padrós F., Golomazou E., Zarza C. (2024). Antiparasitic Approaches and Strategies in European Aquaculture, with Emphasis on Mediterranean Marine Finfish Farming: Present Scenarios and Future Visions. Rev. Aquac..

[16] Wassmuth R.M., de Jongh E.J., Uhland F.C., Reid-Smith R.J.J., Robertson K., Otto S.J.G.J.G. (2024). Factors Associated with Disease in Farmed and Wild Salmonids Caused by *Tenacibaculum maritimum*: A Scoping Review. Front. Aquac..

[17] Juárez-Cortés M.Z., Vázquez L.E.C., Díaz S.F.M., Cardona Félix C.S. (2024). *Streptococcus iniae* in Aquaculture: A Review of Pathogenesis, Virulence, and Antibiotic Resistance. Int. J. Vet. Sci. Med..

[18] Alsheikh-Hussain A.S., Ben Zakour N.L., Forde B.M., Rudenko O., Barnes A.C., Beatson S.A., Silayeva O. (2022). A High-Quality Reference Genome for the Fish Pathogen *Streptococcus iniae*. Microb. Genom..

[19] Liu Y.-L., Chen X.-W., Tian S.-Q., Tan X.-H., Peng B. (2024). Edwardsiella Tarda Attenuates Virulence upon Oxytetracycline Resistance. J. Proteome Res..

[20] Kamiyama S., Kuriyama A., Hashimoto T. (2019). Edwardsiella Tarda Bacteremia, Okayama, Japan, 2005–2016. Emerg. Infect. Dis..

[21] Avendaño-Herrera R., Toranzo A.E., Magariños B. (2006). Tenacibaculosis Infection in Marine Fish Caused by *Tenacibaculum maritimum*: A Review. Dis. Aquat. Organ.

[22] Milijasevic M., Veskovic-Moracanin S., Babic Milijasevic J., Petrovic J., Nastasijevic I. (2024). Antimicrobial Resistance in Aquaculture: Risk Mitigation within the One Health Context. Foods.

[23] Mohammed E.A.H., Kovács B., Kuunya R., Mustafa E.O.A., Abbo A.S.H., Pál K. (2025). Antibiotic Resistance in Aquaculture: Challenges, Trends Analysis, and Alternative Approaches. Antibiotics.

[24] Gonzalez-Avila L.U., Loyola-Cruz M.A., Hernández-Cortez C., Bello-López J.M., Castro-Escarpulli G. (2021). Colistin Resistance in *Aeromonas* Spp. Int. J. Mol. Sci..

[25] Carusi J., Kabuki D.Y., de Seixas Pereira P.M., Cabral L. (2024). *Aeromonas* spp. in Drinking Water and Food: Occurrence, Virulence Potential and Antimicrobial Resistance. Food Res. Int..

[26] Saxena S., Spaink H.P., Forn-Cuní G. (2021). Drug Resistance in Nontuberculous Mycobacteria: Mechanisms and Models. Biology.

[27] Castro R.A.D., Borrell S., Gagneux S. (2020). The Within-Host Evolution of Antimicrobial Resistance in Mycobacterium Tuberculosis. FEMS Microbiol. Rev..

[28] Deng Y., Xu L., Chen H., Liu S., Guo Z., Cheng C., Ma H., Feng J. (2020). Prevalence, Virulence Genes, and Antimicrobial Resistance of *Vibrio* Species Isolated from Diseased Marine Fish in South China. Sci. Rep..

[29] Morshdy A.E.M.A., El Galil G.M.A., El Bayomi R.M., Al Ali A., Mohamed R.E., Shosha A.M., Darwish W.S. (2025). Prevalence of Multidrug-Resistant *Vibrio* Species in Fish in a Reduction Trial Using Lemon Juice and Sesame Oil. Open Vet. J..

[30] Suzuki S., Hoa P.T.P. (2012). Distribution of Quinolones, Sulfonamides, Tetracyclines in Aquatic Environment and Antibiotic Resistance in Indochina. Front. Microbiol..

[31] Done H.Y., Venkatesan A.K., Halden R.U. (2015). Does the Recent Growth of Aquaculture Create Antibiotic Resistance Threats Different from Those Associated with Land Animal Production in Agriculture?. AAPS J..

[32] Gutiérrez-Pacheco M.M., Gracia-Valenzuela M.H., Ortega-Ramirez L.A., Vázquez-Armenta F.J., Leyva J.M., Ayala-Zavala J.F., Chávez-Almanza A.F. (2026). Joining Forces Against Antibiotic Resistance in Aquaculture: The Synergism Between Natural Compounds and Antibiotics. Antibiotics.

[33] Rodríguez-Martínez J.M., Machuca J., Cano M.E., Calvo J., Martínez-Martínez L., Pascual A. (2016). Plasmid-Mediated Quinolone Resistance: Two Decades On. Drug Resist. Updat. Rev. Comment. Antimicrob. Anticancer Chemother..

[34] Partridge S.R., Kwong S.M., Firth N., Jensen S.O. (2018). Mobile Genetic Elements Associated with Antimicrobial Resistance. Clin. Microbiol. Rev..

[35] Pepi M., Focardi S. (2021). Antibiotic-Resistant Bacteria in Aquaculture and Climate Change: A Challenge for Health in the Mediterranean Area. Int. J. Environ. Res. Public Health.

[36] Letchumanan V., Chan K.-G., Lee L.-H. (2014). Vibrio Parahaemolyticus: A Review on the Pathogenesis, Prevalence, and Advance Molecular Identification Techniques. Front. Microbiol..

[37] Paredes-Trujillo A., Cano Rufino L., Hernández-Pérez A. (2024). Parasites of Veterinary Importance of Ornamental Fish Commercialized in Mexico. Vet. Parasitol. Reg. Stud. Rep..

[38] Au-Yeung C., Tsui Y.-L., Choi M.-H., Chan K.-W., Wong S.-N., Ling Y.-K., Lam C.-M., Lam K.-L., Mo W.-Y. (2025). Antibiotic Abuse in Ornamental Fish: An Overlooked Reservoir for Antibiotic Resistance. Microorganisms.

[39] Sivaraman G.K., Rajan V., Vijayan A., Elangovan R., Prendiville A., Bachmann T.T. (2021). Antibiotic Resistance Profiles and Molecular Characteristics of Extended-Spectrum Beta-Lactamase (ESBL)-Producing *Escherichia coli* and *Klebsiella pneumoniae* Isolated from Shrimp Aquaculture Farms in Kerala, India. Front. Microbiol..

[40] Debnath P.P., Chokmangmeepisarn P., Papadopoulou A., Coyle N.M., Baker-Austin C., van Aerle R., Bass D., Tyler C.R., Rodkhum C. (2025). Diversity and Antimicrobial Resistance among Bacterial Isolates from Finfish Aquaculture in Thailand. BMC Vet. Res..

[41] The Economic Case for One Health. https://www.fao.org/one-health/highlights/the-economic-case-for-one-health/en.

[42] Manaia C.M., Rocha J., Scaccia N., Marano R., Radu E., Biancullo F., Cerqueira F., Fortunato G., Iakovides I.C., Zammit I. (2018). Antibiotic Resistance in Wastewater Treatment Plants: Tackling the Black Box. Environ. Int..

[43] India-Legitquest Environment (Protection) Amendment Rules, 2019. https://www.legitquest.com/act/environment-protection-amendment-rules-2019/94B3.

[44] FAO, UNEP, WHO, WOAH (2022). One Health Joint Plan of Action, 2022–2026. Working Together for the Health of Humans, Animals, Plants and the Environment.

[45] von Wintersdorff C.J.H., Penders J., van Niekerk J.M., Mills N.D., Majumder S., van Alphen L.B., Savelkoul P.H.M., Wolffs P.F.G. (2016). Dissemination of Antimicrobial Resistance in Microbial Ecosystems through Horizontal Gene Transfer. Front. Microbiol..

[46] Doyle C., Wall K., Fanning S., McMahon B.J. (2025). Making Sense of Sentinels: Wildlife as the One Health Bridge for Environmental Antimicrobial Resistance Surveillance. J. Appl. Microbiol..

[47] Young K.M., Isada M.J., Reist M., Uhland F.C., Sherk L.M., Carson C.A. (2023). A Scoping Review of the Distribution and Frequency of Extended-Spectrum β-Lactamase (ESBL)-Producing Enterobacteriaceae in Shrimp and Salmon. Epidemiol. Infect..

[48] Rose M. (2026). Risks Associated with Dietary Exposure to Contaminants from Foods Obtained from Marine and Fresh Water, Including Aquaculture. Int. J. Environ. Res. Public Health.

[49] Lulijwa R., Rupia E.J., Alfaro A.C. (2020). Antibiotic Use in Aquaculture, Policies and Regulation, Health and Environmental Risks: A Review of the Top 15 Major Producers. Rev. Aquac..

[50] Hall-Spencer J.M., Harvey B.P. (2019). Ocean Acidification Impacts on Coastal Ecosystem Services Due to Habitat Degradation. Emerg. Top. Life Sci..

[51] Makarova M.A. (2018). Pathogenic Potential of Commensal *Escherichia coli* Isolated from Adults in Saint Petersburg. Russ. J. Infect. Immun..

[52] Ahmed I., Ishtiyaq S., Sayed S.F. (2025). An Overview on Understanding the Major Bacterial Fish Diseases in Freshwater Salmonids. Front. Aquac..

[53] Singh B.K., Thakur K., Kumari H., Mahajan D., Sharma D., Sharma A.K., Kumar S., Singh B., Pankaj P.P., Kumar R. (2024). A Review on Comparative Analysis of Marine and Freshwater Fish Gut Microbiomes: Insights into Environmental Impact on Gut Microbiota. FEMS Microbiol. Ecol..

[54] Abualreesh M.H.H. (2026). Effects of Probiotics, Prebiotics, and Synbiotics on Immune Function, Disease Resistance, Digestive Health, and Stress Management in Fish Culture. Front. Mar. Sci..

[55] Ching C., Zaman M.H., Wirtz V.J. (2024). Evaluation of Surveillance Strategies of Antimicrobial Consumption in Animals. Antibiotics.

[56] Chen W., Zeng Y., Zheng J., Wang J., Gu W., Li M., Cheng Z., Qian J., Zhang X., Kabali E. (2026). Evaluation of Antimicrobial Resistance Governance Across 193 Countries to Inform the 2026 Global Action Plan Update. Nat. Med..

[57] Li F., Liu K., Bao Y., Li Y., Zhao Z., Wang P., Zhan S. (2024). Molecular Level Removal of Antibiotic Resistant Bacteria and Genes: A Review of Interfacial Chemical in Advanced Oxidation Processes. Water Res..

[58] Hart A., Warren J., Wilkinson H., Schmidt W. (2023). Environmental Surveillance of Antimicrobial Resistance (AMR), Perspectives from a National Environmental Regulator in 2023. Eurosurveillance.

[59] Balcázar J.L. (2025). Wastewater-Based Epidemiology as a Complementary Tool for Antimicrobial Resistance Surveillance: Overcoming Barriers to Integration. BioEssays News Rev. Mol. Cell. Dev. Biol..

[60] UN Environment Programme Bracing for Superbugs: Strengthening Environmental Action in the One Health Response to Antimicrobial Resistance|UNEP—UN Environment Programme. https://www.unep.org/resources/superbugs/environmental-action.

[61] Caputo A., Bondad-Reantaso M.G., Karunasagar I., Hao B., Gaunt P., Verner-Jeffreys D., Fridman S., Dorado-Garcia A. (2023). Antimicrobial Resistance in Aquaculture: A Global Analysis of Literature and National Action Plans. Rev. Aquac..

[62] Tegegne H.A., Bogaardt C., Collineau L., Cazeau G., Lailler R., Reinhardt J., Freeth F.T.A., Taylor E., Prada J.M., Hénaux V. (2023). OH-EpiCap: A Semi-Quantitative Tool for the Evaluation of One Health Epidemiological Surveillance Capacities and Capabilities. Front. Public Health.

[63] Randeni R.A.S.B., Badanasinghe C.N., Pathirannahalage S.-C.N. (2026). Tracking Environmental Antimicrobial Resistance: Isolation and Antimicrobial Susceptibility Patterns of *E. coli* across Hospital, Livestock, and Community Settings in Gampaha, Sri Lanka. BMC Microbiol..

[64] Singer A.C., Shaw H., Rhodes V., Hart A. (2016). Review of Antimicrobial Resistance in the Environment and Its Relevance to Environmental Regulators. Front. Microbiol..

[65] Deng Y., Tan A., Zhao F., Wang F., Gong H., Lai Y., Huang Z. (2025). Global Distribution of Antimicrobial Resistance Genes in Aquaculture. One Health Adv..

[66] Akter L., Hasan N.A., Rahman M., Forajy N., Haque M.M. (2026). Antimicrobial Resistance in Shrimp Aquaculture: Pathways, Ecosystem Risks, and Policy Responses. Environ. Chall..

[67] Tuševljak N., Dutil L., Rajić A., Uhland F.C., McClure C., St-Hilaire S., Reid-Smith R.J., McEwen S.A. (2013). Antimicrobial Use and Resistance in Aquaculture: Findings of a Globally Administered Survey of Aquaculture-Allied Professionals. Zoonoses Public Health.

[68] Calero-Cáceres W., Marti E., Olivares-Pacheco J., Rodriguez-Rubio L. (2022). Editorial: Antimicrobial Resistance in Aquatic Environments. Front. Microbiol..

[69] Çağatay I.T. (2023). Bacteriophage Applications in Aquaculture. Isr. J. Aquac.-Bamidgeh.

[70] Melo L.D.R., Oliveira H., Pires D.P., Dabrowska K., Azeredo J. (2020). Phage Therapy Efficacy: A Review of the Last 10 Years of Preclinical Studies. Crit. Rev. Microbiol..

